# Optimized Interfaces in Anti-Perovskite Electrolyte-Based Solid-State Lithium Metal Batteries for Enhanced Performance

**DOI:** 10.3389/fchem.2021.786956

**Published:** 2021-12-23

**Authors:** Pengcheng Yu, Yu Ye, Jinlong Zhu, Wei Xia, Yusheng Zhao

**Affiliations:** ^1^ Academy for Advanced Interdisciplinary Studies and Department of Physics, Southern University of Science and Technology, Shenzhen, China; ^2^ Shenzhen Key Laboratory of Solid State Batteries, Guangdong Provincial Key Laboratory of Energy Materials for Electric Power, Southern University of Science and Technology, Shenzhen, China

**Keywords:** lithium metal anode, solid-state batteries, interface optimization, *in-situ* solidification, anti-perovskite electrolyte

## Abstract

Solid-state lithium metal batteries have attracted broad interest as a promising energy storage technology because of the high energy density and enhanced safety that are highly desired in the markets of consumer electronics and electric vehicles. However, there are still many challenges before the practical application of the new battery. One of the major challenges is the poor interface between lithium metal electrodes and solid electrolytes, which eventually lead to the exceptionally high internal resistance of the cells and limited output. The interface issue arises largely due to the poor contact between solid and solid, and the mechanical/electrochemical instability of the interface. In this work, an *in situ* “welding” strategy is developed to address the interfacial issue in solid-state batteries. Microliter-level of liquid electrolyte is transformed into an organic–inorganic composite buffer layer, offering a flexible and stable interface and promoting enhanced electrochemical performance. Symmetric lithium–metal batteries with the new interface demonstrate good cycling performance for 400 h and withstand the current density of 0.4 mA cm^−2^. Full batteries developed with lithium–metal anode and LiFePO_4_ cathode also demonstrate significantly improved cycling endurance and capacity retention.

## Introduction

Although the energy density of lithium-ion batteries (LIBs) has increased continually over the past 30 years, LIBs are still difficult to meet the future requirement of long endurance in energy storage and transportation (Yoshio et al., 2009; Zubi et al., 2018). Because of the limited specific energy of the electrode materials, the energy density of LIBs has reached the bottleneck (Manthiram, 2017). To increase the energy density of lithium batteries, it is necessary to construct a new chemical system. As one of the most promising anode materials, lithium metal demonstrates a marvelous theoretical capacity (3,860 mAh g^−1^) and the lowest electrochemical potential (−3.04 V to standard hydrogen electrode) (Cheng et al., 2017; Krauskopf et al., 2020). Therefore, lithium metal has been reported as one of the most promising candidates for the next-generation battery (Cheng et al., 2017). However, it is hard to form a stable and dense SEI layer on the lithium metal anodes of cells with liquid electrolytes due to the continuous reactions between lithium metal and the liquid electrolytes (Cheng et al., 2017; Krauskopf et al., 2020; Han et al., 2021). Moreover, inhomogeneous lithium deposition would trigger dendrite or dead lithium, and eventually cause low Coulombic efficiency or even serious safety issues (Cheng et al., 2017; Zubi et al., 2018; Lu et al., 2019). As a result, the practical application of high-energy-density lithium metal anode in reversible batteries has not been be achieved yet.

On the other hand, remarkable progress has been made in solid ionics recently, which provide a promising key to unlock the lithium metal anode (Cheng et al., 2017; Schnell et al., 2018). Replacing the liquid electrolytes with solid-state electrolytes (SSEs) could limit dendrite penetration by providing a rigid physical barrier (Schnell et al., 2018). The Li transfer number is generally higher in SSEs than that in liquid electrolytes, which helps to alleviate concentration gradient and contribute to homogeneous Li deposition (Cheng et al., 2017). Moreover, the solid electrolytes are free of leakage and are non-flammable, providing much more safety insurance than the flammable liquid electrolytes (Dunn et al., 2011; Xu et al., 2014; Krauskopf et al., 2020). The most studied SSEs include polymers, oxides, and sulfide electrolytes (Agrawal and Pandey, 2008; Janek and Zeier, 2016; Luo et al., 2016; Zhang et al., 2019). Some of them have demonstrated ionic conductivities that are comparable with liquid electrolytes (Zhang et al., 2019), paving the way for the application in batteries. However, there exist some obstacles in the practical application and the interface instability would be a major one (Janek and Zeier, 2016; Pervez et al., 2019). Most of the high-conducting SSEs possess narrow electrochemical windows and poor compatibility toward electrode materials, particularly toward the lithium metal anode (Pervez et al., 2019). As a result, an interlayer with high ionic resistance would form between the SSEs and lithium metal anode (Park et al., 2018; Pervez et al., 2019).

Recently, a new type of oxyhalide electrolytes (Li_3_OX, X = Cl, Br) with interesting anti-perovskite structures have been developed and displayed intrinsic stability toward the lithium metal anode and high ionic conductivity (10^−3^ S/cm level at room temperature) (Zhao and Daemen, 2012; Lu et al., 2020; Deng et al., 2021). Later, the proton-rich derivatives Li_2_OHX (X = Cl, Br, I) have also been synthesized and showed higher purity and milder synthesis process (Hood et al., 2016; Li et al., 2016; Xiao et al., 2021). The Li_2_OHX (X = Cl, Br, I) electrolytes possess high lithium content and a similar crystal structure to Li_3_OX but with higher phase stability and can be prepared from low-price starting materials (Hood et al., 2016; Li et al., 2016; Koedtruad et al., 2020; Lu et al., 2020; Deng et al., 2021; Guo et al., 2021). They have also displayed remarkable chemical and electrochemical stabilities toward lithium metals even at elevated temperatures (Guo et al., 2021; Lai et al., 2021). The ionic conductivities of these derivatives are lower than that of the parent Li_3_OX, but still reach 10^−3^ S/cm at 100°C (Hood et al., 2016; Li et al., 2016; Xiao et al., 2021). More recently, by utilizing the low-melting point character of these electrolytes (Lai et al., 2021), all-solid-state batteries with LiNi_0.33_Mn_0.33_Co_0.33_O_2_ cathodes and Li_4_Ti_5_O_12_ or graphite anodes were fabricated through an energy-efficient melt-infiltration method (Xiao et al., 2021). The battery exhibited compact assembly of cathode, electrolyte and anode layers, and displayed promising electrochemical performances. Given the high stability toward the lithium metal, a Li_2_OHCl electrolyte coating layer can be adopted in a tradition garnet electrolyte system (Lai et al., 2021), all-solid-state batteries with lithium metal anodes can also be anticipated (Lai et al., 2021). However, considering the low melting point of lithium metal, the above melt-infiltration method would be infeasible. Melting Li metal in the assembling process has been reported to provide a better anode–SSE interface contact because melted Li metal fills the uneven surface of the electrolyte (Wang et al., 2017; Wu et al., 2018; Yang et al., 2019). However, Li metal cannot spread on the untreated surface of an intrinsic lithiophobic SSE material. Applying pressure to the assembly of electrode and electrolyte was generally used to pursue good contact between the different layers (Zhang et al., 2017). Constructing a polymer–oxide–polymer sandwich composite electrolyte can also improve the interface contact (Lu et al., 2019; Li et al., 2021). However, due to the poor mobility of the solid material, the interface of the lithium anode electrolyte and the SSE pellet is unavoidable to become solid–solid point contact (Janek and Zeier, 2016; Zhang et al., 2017; Lu et al., 2019; Li et al., 2021). The limited contact area blocks the Li^+^ transport channel and significantly increases the interface impedance (Lu et al., 2019; Li et al., 2021). In cycling of the cell, dreadful interface physical contact aggravates the uneven deposition of the Li^+^, which eventually leads to dendrite growth and battery failure (Janek and Zeier, 2016; Xu et al., 2018; Lu et al., 2019; Xiao et al., 2020). Therefore, it is necessary to develop a new strategy to fulfill a stable contact between the electrolytes and electrodes.

In this work, the interfacial issue in solid-state batteries is addressed by an *in situ* “welding” strategy. Specifically, the interface between anode and the solid-state electrolyte is wetted by microliter-level of liquid electrolyte during cell assembling, which is transformed into an organic-inorganic composite buffer layer. In the *in situ* solidified layer, the soft organic component connects the lithium metal anode with the solid-state electrolytes, buffers the volume change of the lithium metal anode during the plating/stripping process, eliminate solid–solid point contact and ensures a regulated Li^+^ flow on the interface; meanwhile, the inorganic component demonstrates much better electrochemical stability offering a flexible and stable interface and promoting enhanced electrochemical performance. Symmetric lithium–metal batteries with the new interface demonstrate good cycling performance for 400 h and withstand the current density of 0.4 mA cm^−2^. Full batteries developed with lithium–metal anode and LiFePO_4_ cathode also demonstrate significantly improved cycling endurance and capacity retention.

## Experiment

### Chemical and Materials

The chemicals and materials used are as follows: lithium hydroxide (LiOH, 99 wt%, Aladdin), lithium chloride (LiCl, 99wt%, Aladdin), N,N-dimethylformamide (DMF, AR, Aladdin), dimethyl sulfoxide (DMSO, AR, Aladdin), 1,3-dioxolane (DOL, AR, Aladdin), tetrahydrofuran (THF, AR, Aladdin), 1,2-dimethoxyethane (DME, AR, Aladdin), acetonitrile (ACN, AR, Aladdin) and dimethyl carbonate (DMC, AR, Aladdin), N-methyl pyrrolidone (NMP, AR, Aladdin), poly (vinylidene fluoride) (PVDF, Mw 400 000, Macklin), lithium bis(tri-fluoromethanesulfonyl) imide (LiTFSI, 99.9 wt%, Macklin), LiPF_6_ (99.9 wt%, Macklin), LiFePO_4_ powder (LFP, 99.9 wt%, MTI Co., Ltd), Super-P (99.9 wt%, MTI Co. Ltd), aluminum foil (MTI Co., Ltd), and Li foil (99.9 wt%, Alfa Aesar).

### Preparation of Li_2_OHCl Powder and Pellet

Li_2_OHCl electrolytes were prepared by mixing appropriate molar ratios of LiOH and LiCl by hand milling and transferred to a nickel crucible. The mixture was then heated to 450°C, held for 4 h, and cooled naturally. Then the product was ground into fine powder. In order to get the pellet form of electrolyte, the obtained 0.15 g Li_2_OHCl powder was pressed into a Φ10 pellet with a mold under 100 MPa and sintered at 290°C for 12 h to form a rigid pellet. All the abovementioned operations were conducted in an Ar-filled glovebox (H_2_O < 0.01 ppm).

### Solvent Compatibility Test

To test the solvent compatibility of Li_2_OHCl, 0.2 g Li_2_OHCl and 2 ml of certain solvents were mixed and stirred for 2 h in an Ar-filled glovebox (H_2_O < 0.01 ppm). After the stirring, the mixture was heated for 12 h in a vacuum to remove the solvent. The dried powder is characterized by X-ray diffraction (XRD) and Fourier transform infrared (FTIR), respectively. It was tested if Li_2_OHCl still demonstrates the same diffraction peak positions in XRD patterns and the absorption peak at the wavenumber of 3,604 cm^−1^ in IR spectra as the intrinsic Li_2_OHCl powder (Supplementary Figures S2 and S3). Based on the results, the solvent compatibility and incompatibility of Li_2_OHCl is evaluated in Supplementary Table S1.

### Characterization

PANalytical Emp3 Diffraction System was used to measure the crystalline of the sample; samples were protected by polyimide film (Kapton™) from the moisture in the air. The morphology was characterized by HITACHI SU8010 field-emission scanning electron microscope (FESEM), and a transfer suitcase was used to isolate samples from the air. The Fourier transform infrared (FTIR) spectra were examined with Thermo Scientific iS50 ATR. X-ray photoelectron spectroscopy (XPS) was performed by ULVAC-PHI PHI 5000 Versaprobe Ⅲ.

### Electrochemical Measurements

The LiFePO_4_ cathode was prepared by casting the slurry containing 60 wt% LiFePO_4_, 15 wt% PVDF, 15 wt% LiTFSI, and 10 wt% Super-P on the aluminum current collector foil, and the cathode foil was coated by a thin layer of polymer (1 M LiTFSI in PEO: PVDF = 1:1) to protect the cathode from electrochemical reactions. CR 2032 coin cells were assembled with lithium metal anode, SSE pellet, and cathode foil. During the cell assembly process, the SSE pellet was wetted with 3 μl of LE (enough to wet the Li–SSE interface), then a lithium foil (Φ6 mm, 100-μm thick) was pressed to the wetted SSE pellet. The customized Swagelok cell was used to conduct the electrochemical impedance spectroscopy (EIS). Autolab PGSTAT 302F performed the electrochemical tests, and LAND CT 2001A performed the cycle test of the batteries at 80°C.

## Result

**FIGURE 1:**
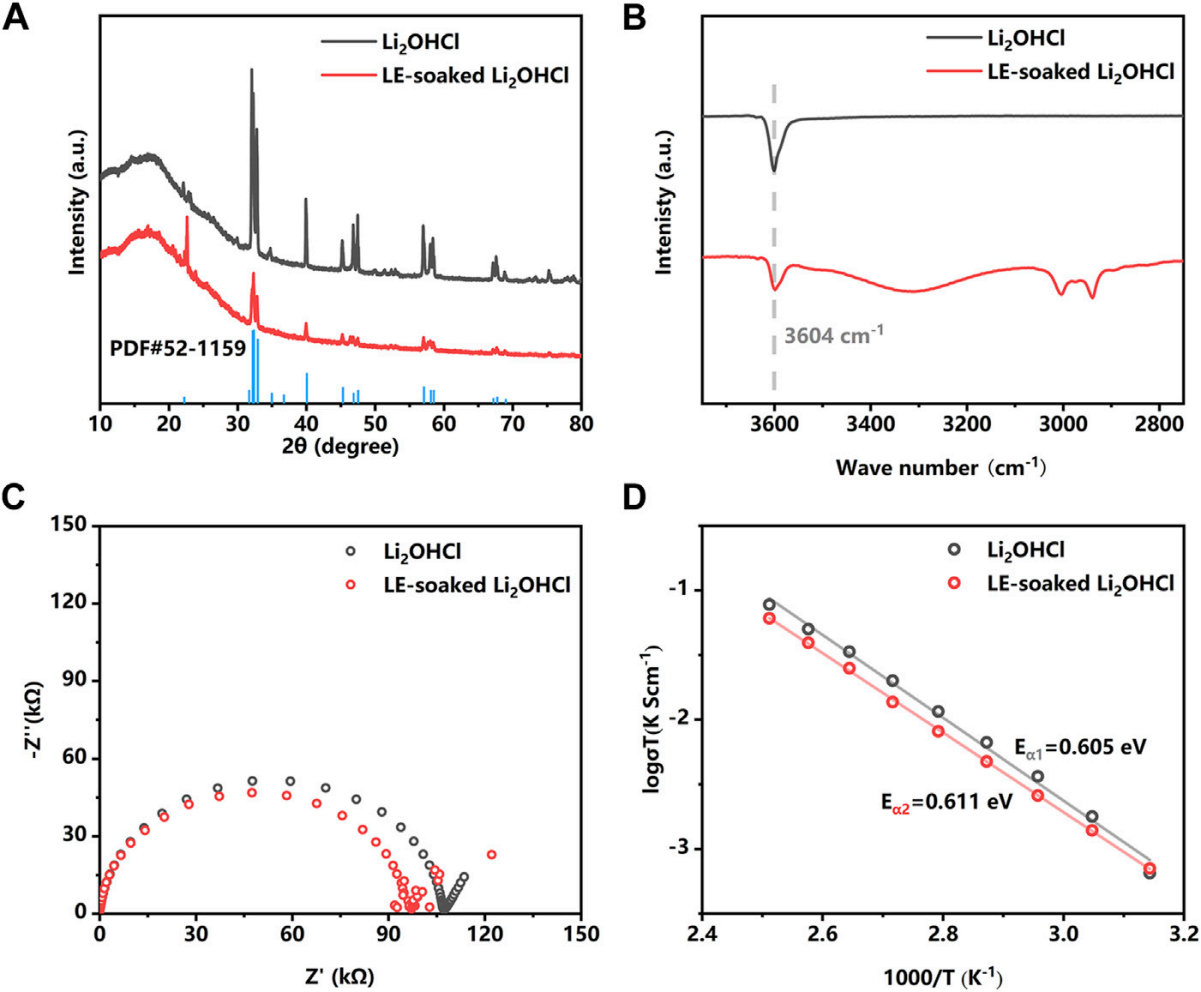
X-ray diffraction (XRD) **(A)**, IR **(B)**, and Nyquist plots **(C)** patterns of the Li_2_OHCl powder before and after the liquid electrolyte (LE) soaking. **(D)** The Arrhenius plots of the Li_2_OHCl and LE-soaked Li_2_OHCl from 45°C to 135°C.

In order to wet the SSE pellet with liquid electrolytes (LEs) to optimize the interface performance, the chemical compatibility of the Li_2_OHCl with solvents need to be investigated. The result of solvent compatibility tests (Supplementary Figures S2 and S3) shows that Li_2_OHCl demonstrates good solvent compatibility to various common battery solvents except high polar solvents such as water, ethanol, DMSO, and DMF. Based on this result, the LE (a solution of 1 M LiPF_6_ in EC:DEC:FEC = 45:45:10 v/v/v) was formulated and further investigated its compatibility with Li_2_OHCl. The XRD patterns of the synthesized Li_2_OHCl powder and the LE soaked Li_2_OHCl powder are shown in Figure 1A. The peak position of Li_2_OHCl powder prepared by the calcining method matches the standard PDF card of the orthorhombic Li_2_OHCl (PDF#52-1159) well. The bulge at about 20°is attributed to the Kapton film. Different intensities of some peaks can be attributed to the transition from the orthorhombic phase to the cubic phase during solvent removing and drying. XRD results reveal that the crystal structure of the Li_2_OHCl does not change after the LE exposure. Moreover, IR spectroscopy (Figure 1B) shows the absorption peak at the wavenumber of 3,604 cm^−1^ in the IR spectra, which can be assigned to the O–H structure in Li_2_OHCl (Supplementary Figure S4). After the soaking of LE, there was no visible shift of the O–H absorption peak of the Li_2_OHCl; the peaks around 3,000 cm^−1^ correspond to the traces of organic solvents remaining in the sample. EIS plots (Figure 1C) reveal that the impedance of the LE-exposed Li_2_OHCl is close to that of the intrinsic Li_2_OHCl, indicating that LE exposure does not deteriorate the ionic conductivity of the Li_2_OHCl. The slight decrease in the impedance of the LE-exposed Li_2_OHCl could be related to the incomplete removal of LE. As shown in Figure 1D, the Arrhenius plots reveal that the activation energy of the Li_2_OHCl before and after the LE-exposure are 0.605 and 0.611 eV, respectively, which indicates the stability of Li_2_OHCl in LE. It is worth noting that the difference in ionic conductivity can be attributed to the residual non-volatile components in the liquid electrolyte.

**FIGURE 2:**
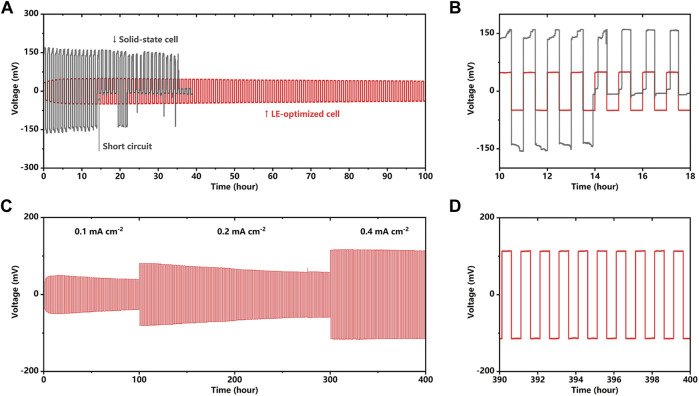
**(A,B)** Galvanostatic cycling of Li/Li_2_OHCl/Li cell with and without LE optimization at a current density of 0.1 mA cm^−2^. **(C,D)** Galvanostatic cycling of LE-optimized Li/Li_2_OHCl/Li cell at current densities of 0.1 mA cm^−2^ (100 h), 0.2 mA cm^−2^ (200 h), and 0.4 mA cm^−2^ (100 h).

Figure 2 depicts the cycling stability of lithium metal symmetric batteries. As shown in Figure 2A, for the cell with a bare SSE pellet, in a current density of 0.1 mA cm^−2^, the overpotential comes to more than 150 mV, which corresponds to a high cell impedance of more than 1,500 Ω cem^−2^. In addition, there is a sudden drop of the overpotential at only the 14th cycle; after that, the cycling becomes unstable until the cell fails after only 37 cycles. The plunged overpotential can be attributed to the growth of the dendrites inside the electrolyte and caused the short circuit. The dendrite deteriorated the interface contact, pierced the SSE pellet, and finally came to another electrode causing the short circuit. In contrast, the cell with a LE-wetted interface exhibits satisfying cycling performance, the overpotential slightly grows in the first 10 cycles, and then stabilizes at a low and stable value of 50 mV. Figure 2B provides a zoomed view of the first 10 cycles, implying the cell’s potential plateau with and without the LE wetting. The cell with a bare SSE pellet demonstrates wedge-shaped voltage plateaus because the Li^+^ transport resistance increases with the deposition progress and results in uneven lithium deposition. Solid–solid point contact and the volume change of the lithium metal anode lead to the uneven lithium plating/stripping and the continued decrease in the effective contact area between the lithium metal anode and SSE pellet. Small contact area corresponding to the increased local current density, which makes the uneven deposition of lithium metal more serious, result in the dendrite growing in the solid-state electrolyte (Supplementary Figure S5). In contrast, the potential curve of the LE-optimized cell shows that the LE-wetting ensures the uniform current distribution on the SSE pellet, thereby effectively inhibiting the growth of the dendrite on the anode and extending the cycle endurance. As shown in Figure 2C, the cell with the LE-wetted interface can cycle in 0.1 mA cm^−2^ for 100 cycles and 0.2 mA cm^−2^ for 200 cycles, respectively. Increasing the current density of the 0.4 mA cm^−2^, the cell still cycles for 100 cycles stably. In the first 280 cycles, the overpotential is reduced with the cycles, which is similar to the interface activation phenomenon in the previous report (Li et al., 2021). The increasing (first 10 cycles) and gradually decreasing (from 10th to 280th cycle) polarization could be attributed to the *in situ* solidification and the long-term compaction of the welding layer. A dense interface layer decreases the interface impedance and lowers the polarization. In Figure 2D, the zoomed view of the overpotential curve from the 390th to 400th cycles shows that even after 390 cycles, the LE-optimized cell still exhibits a low and stable plating/stripping overpotential, which indicates that the cycle stability of cell with LE wetting under a high current density and the interface exhibit a highly efficient and stable lithium-ion transport mechanism.

**FIGURE 3:**
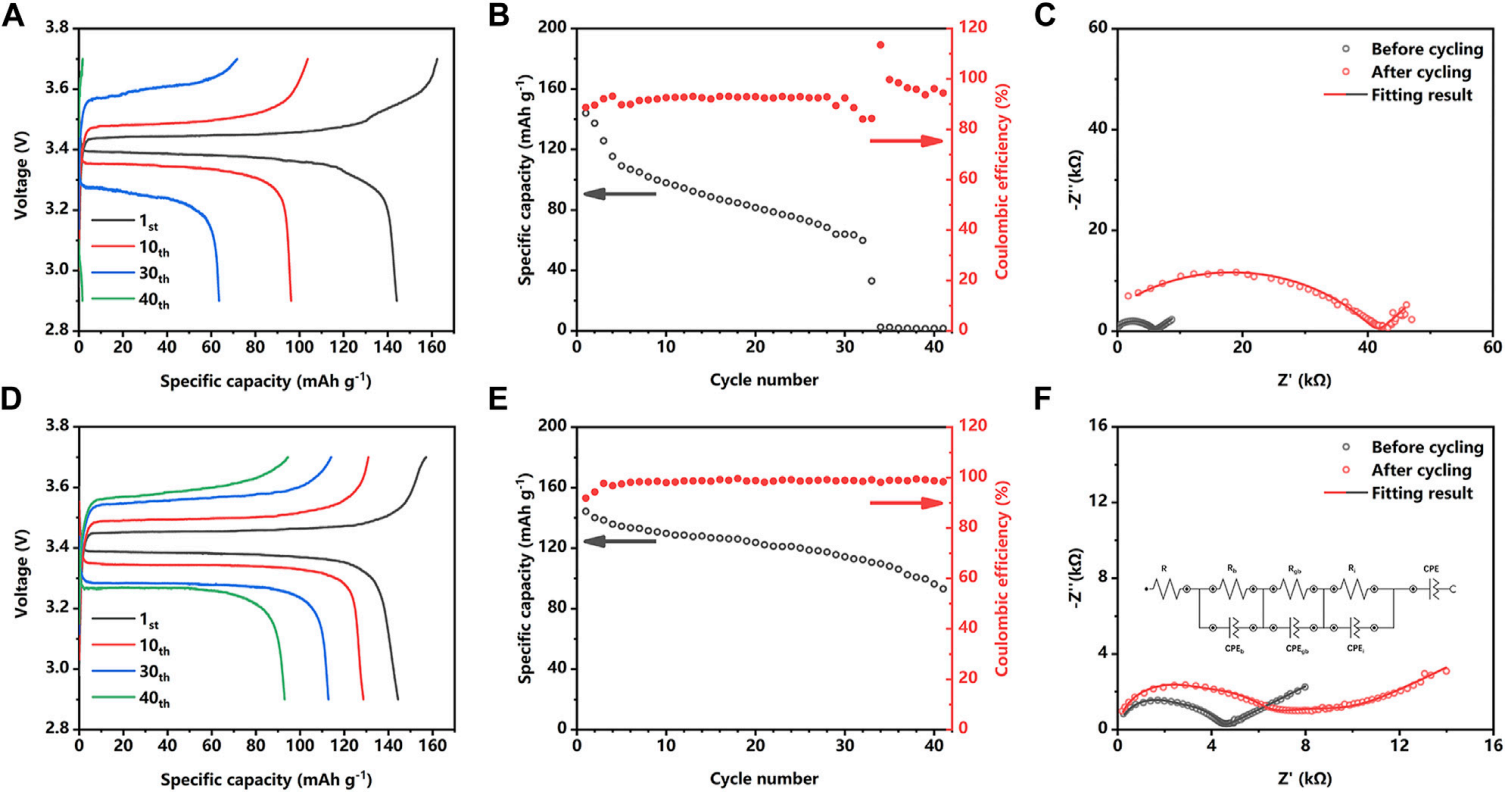
Voltage profiles **(A)** and cycling performance **(B)** of a Li/Li_2_OHCl/LFP cell without LE optimization at 0.2C. Nyquist plots of a Li/Li_2_OHCl/LFP cell with **(F)** and without **(C)** LE optimization at 0.2C before and after cycling. Voltage profiles **(D)** and cycling performance **(E)** of an LE optimization Li/Li_2_OHCl/LFP cell at 0.2C.

The practical performance of the LE-optimization strategy is further evaluated in the Li | Li_2_OHCl | LiFePO_4_ system in coin cells. As shown in Figures 3A, B, the unoptimized Li/LFP cell exhibits a capacity of about 145.4 mAh g^−1^ in the first cycle; however, most of the capacity quickly decayed in less than 35 cycles. Figure 3C reveals that the Coulombic efficiency of the unoptimized Li/LFP cell is only about 90% in the first 20 cycles. It is worth noting that unstable Coulombic efficiency appears in the 29th cycle. The fast capacity decay could be attributed to the irreversible electrochemical reaction of the electrode material during the stripping process, such as the formation of the dendrite and short circuit. In contrast, as shown in Figures 3D, E, the LE-optimized Li/LFP cell exhibits a stable voltage platform and lower overpotential during the plating/stripping process, demonstrating a significantly improved capacity of 144.8 mAh g^−1^ in the first cycle and maintaining 130.6 mAh g^−1^ after 10 cycles. After 40 cycles, the LE-optimized Li/LFP cell still exhibits a capacity of 97 mAh g^−1^. As shown in Figure 3E, the improved capacity retention corresponds to the high Coulombic efficiency, which remains stable after three cycles and keeps higher than 95% in all cycling endurance.

Figures 3C, D depict the evolution of the cell impedance with cycling. Except for the experimental data, the simulated spectra are based on an equivalent circuit comprising an R_b_, R_gb_, and R_i_ associated with the bulk impedance, grain boundary impedance, and the interface impedance, respectively. In Figure 3C, the unoptimized Li/LFP cells exhibit a total impedance of 6.1 kΩ before cycling; however, after 40 cycles, the total impedance dramatically increased to 43 kΩ. According to the results of the simulated spectra, the interface impedance contributes most of the increase of the total impedance, which reveals the occurred interface deterioration such as poor physical contact and dead lithium accumulation. In comparison (Figure 3F), the impedance of LE-optimized Li/LFP cell increases from about 4.7 to 7.9 kΩ after 40 cycles, which corresponds to the growing overpotential of the LE-optimized Li/LFP cell in Figure 3D. The result of the simulated spectra reveals that the interface impedance (R_i_) increases slightly. Although the LE-wetting slightly increases the interface impedance, a stable interface is formed during the following cycles and ensures the cycle stability, thereby improving the cycle endurance and capacity retention.

## Discussion

**FIGURE 4:**
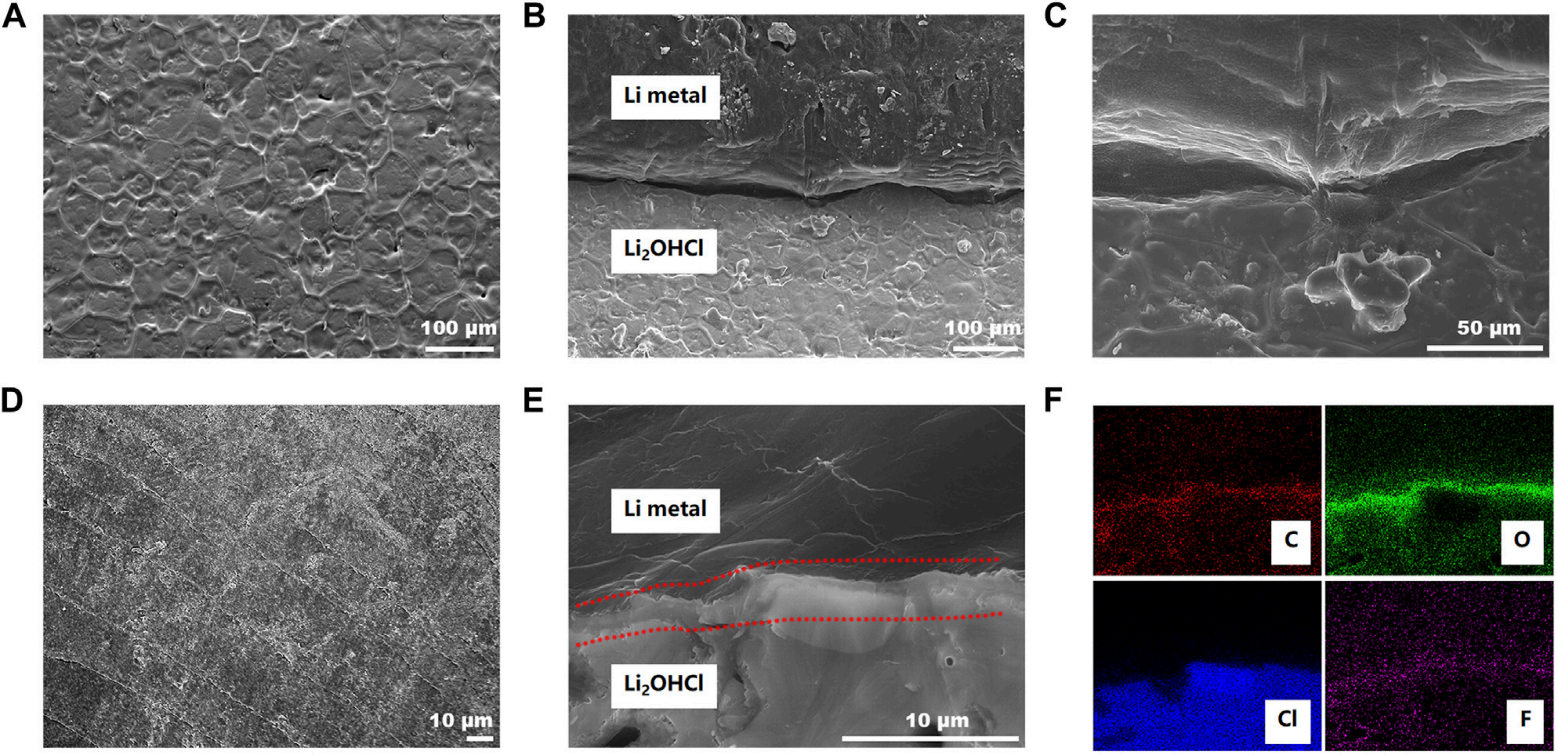
The morphology of the surface **(A)** and cross-sectional **(B,C)** of the Li_2_OHCl pellet after galvanostatic cycling for 20 h in a Li/Li2OHCl/Li cell without LE optimization. The morphology of the surface (D) and cross-sectional (E) of the Li_2_OHCl pellet after galvanostatic cycling for 20 h in an Li/Li2OHCl/Li cell with LE optimization. **(F)** Energy-dispersive X-ray spectroscopy (EDS) mapping images correlated to the figure **(E)** of the carbon, oxygen, chlorine, and fluorine in red, green, blue, and purple, respectively.

The front and cross-sectional of the SSE pellets in lithium metal symmetric cells after 20 cycles were characterized by FESEM to study the mechanism of LE-optimization. As Figure 4A shows, the front surface of the “bare” SSE pellet shows the visible unevenness; the interconnected ridge structure can be found on the surface. The unique morphology could be attributed to the sintering process of the electrolyte particles during the pressing–annealing process. From the cross-sectional view, Figures 4B, C show a gap of about 10 μm between the lithium metal anode and the SSE pellet. Even in contact with a soft material, lithium metal, the SSE pellet with an uneven surface cannot form a good interface contact with the anode. Unsatisfactory interface contact is precisely the point contact of different solid materials, the gaps between the uneven part of the pellet, and the lithium metal, and the voids generated by uneven lithium deposition during the plating and stripping process. In the cell system, even the SSE pellet is relatively flat, and only the ridge of the pellet can contact lithium metal, which significantly decreases the effective contact area between the lithium metal anode and the SSE pellet, and result in a narrow Li^+^ transport channel. In Figure 4D, the surface of the LE-wetted SSE pellet shows a different morphology from that of the “bare” SSE. The surface is flat, and the parallel linear scratches can be attributed to the damage during the removal of the lithium metal anode in the sample preparation process. The front-view picture of the LE-wetted SSE pellet indicates that a flat and soft interface layer was *in situ* formed on the surface after the wetting of the LE. In Figure 4E, the cross-sectional view of the LE-wetted interface shows a 2-μm-thick interface layer “welding” the lithium metal anode and the SSE pellet. The physical contact is improved so much that there is no visible gap between them. The energy-dispersive X-ray spectroscopy (EDS) mapping of C, O, Cl, and F associated with Figure 4E is in Figure 4F. The EDS pictures show that there are much higher C and O containing the interface layer. Noteworthily, the presence of F in the interface layer implies that the layer contains organic and inorganic components, which indicates that the LE was converted into a soft interface layer.

**FIGURE 5:**
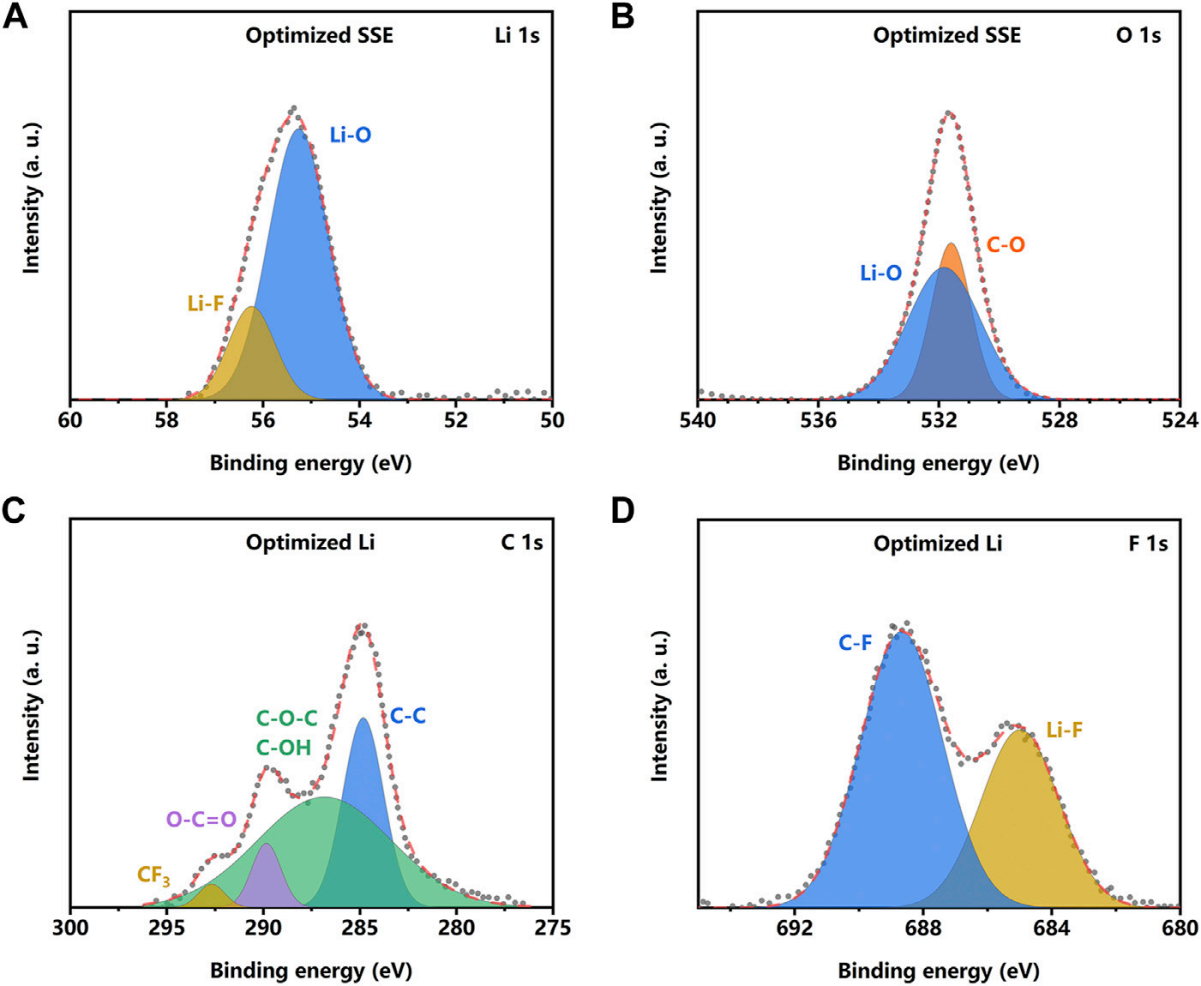
X-ray photoelectron spectroscopy (XPS) data and simulated peaks of Li 1s **(A)** and O 1s **(B)** of the solid-state electrolyte (SSE) surface in a symmetric cell with LE-optimization. XPS data and simulated peaks of C 1s **(C)** and F 1s **(D)** of the Li metal surface in a symmetric cell with LE optimization.

X-ray photoelectron spectroscopy (XPS) was conducted to examine the chemical composition of the surface on the SSE pellet in lithium metal symmetric cells. The XPS result (Supplementary Figure S6) reveals that there are signals of Li, Cl, C, and O on the surface of the bare SSE pellet. As for the LE-wetted SSE pellet, the signal of F can be detected except for the abovementioned elements. The data of Li 1s and O 1s reveals that there are Li_2_OHCl (Li 1s 55 eV, O 1s 531.8 eV), LiOH (Li 1s 54.7 eV, O 1s 531.2 eV), and LiCl (Cl 2p 200.1 eV) (Supplementary Figure S7) on the surface of the bare SSE pellet. The Li_2_OHCl will react with liquid lithium metal at 195°C and forms the SEI layer (Hood et al., 2016; Li et al., 2016; Xiao et al., 2021). In this work, the XPS result reveals that a similar reaction can also occur between the Li_2_OHCl and lithium metal. The newly formed inorganic interface layer is the ionic conductor, which inhibits the further electrochemical reaction and stabilizes the interface. However, the low ionic conductivity increases the Li^+^ transport resistance and the interface impedance, resulting in unsatisfying electrochemical performance. In contrast, the data of Li 1s and F 1s (Figures 5A, D) indicates that the LiF appears in the composite layer, which originates from the reaction between Li metal and liquid electrolyte. The O 1s, C 1s, and F 1s data (Figures 5B–D) indicate that the liquid electrolyte is converted into composite organic and inorganic components. The XPS result reveals that the *in situ* formed organic–inorganic composite layer possesses a similar structure to the SEI layer in lithium metal batteries with the liquid electrolyte (Gao et al., 2019; Shadike et al., 2021). Fluorine-rich polymer constitutes the organic layer; LiF and LiCO_3_ are the main components of the inorganic part.

**FIGURE 6:**
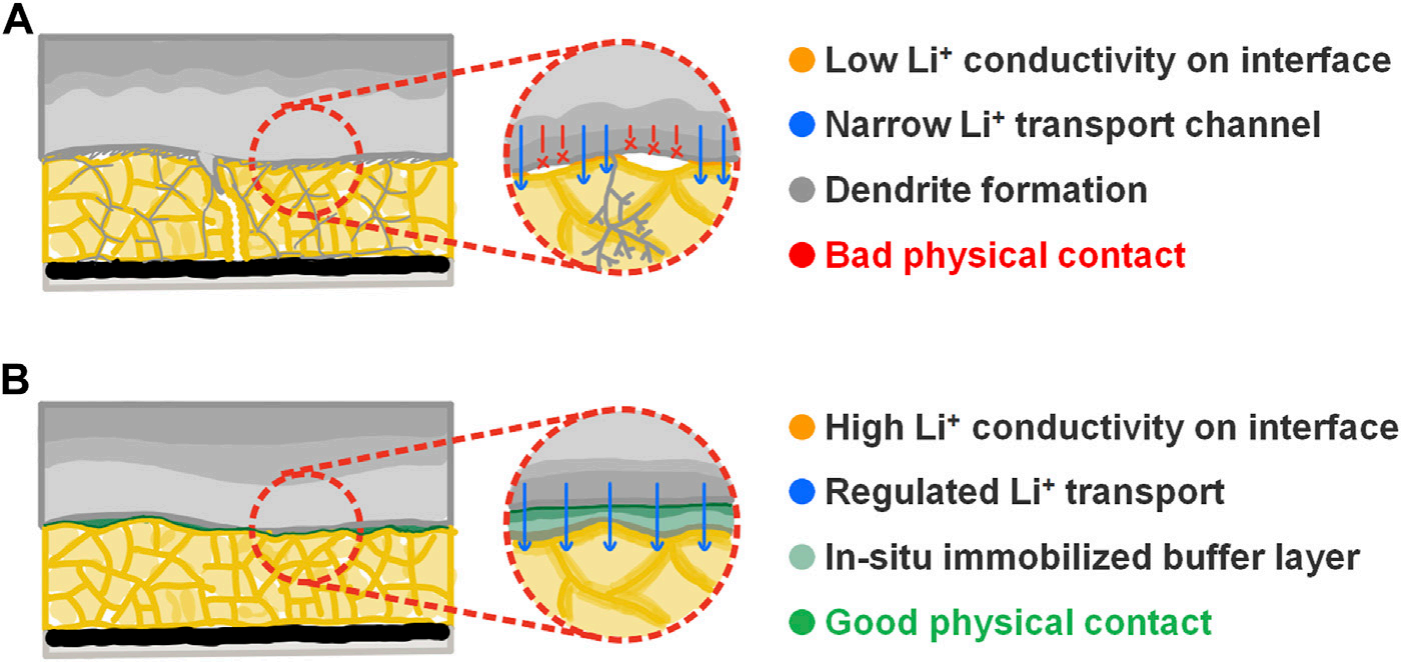
**(A)** Schematic illustrating the bad interface contact between lithium metal anode and the Li_2_OHCl SSE pellet. **(B)** Schematic illustrating the improved interface contact between lithium metal anode and the Li_2_OHCl SSE pellet by the *in situ* solidification reaction of the liquid electrolyte.

The performance of traditional lithium metal batteries with SSE is limited by bad physical and subsequent battery degradation (Figure 6A). Our previous experiments reveal that wetting SSE pellet with microliter-level liquid electrolyte can significantly improve the electrochemical performance of lithium metal anode. As shown in Figure 6B, the schematic shows that after the cell assembly, the LE wets the SSE pellet and fills the gap between the lithium metal anode and the SSE pellet; then with the cycle going on, the LE solidified on the interface and forms an organic–inorganic composite buffer layer after a series of chemical and electrochemical reactions. In the *in situ* solidified layer, the soft organic component welds the lithium metal anode and the SSE pellet together, buffers the volume change of the lithium metal anode during the plating/stripping process, eliminates the solid–solid point contact, and ensures a regulated Li^+^ flow on the interface; meanwhile, compared with the LiCl-Li_2_O SEI (Hood et al., 2016; Li et al., 2016; Xiao et al., 2021), the formation of LiF demonstrates much better electrochemical stability and higher ionic conductivity, so that the inorganic layer enables a low interface impedance while maintaining electrochemical stability.

In summary, we developed a simple *in situ* solidification strategy to improve the interface performance of the solid-state lithium metal battery by “welding” the lithium metal anode and the SSE pellet. First, the solvent compatibility of the Li_2_OHCl is investigated; then, the anode-SSE interface is wetted by a small amount of LE *in situ* to form an inorganic–organic composite welding layer and improve the interface physical and electrochemical performance. Specifically, the solvent compatibility of the Li_2_OHCl is investigated; then, a small amount of LE is added to the SSE–anode interface *in situ* to form a buffer layer and improve the interface physical and electrochemical performance. The consequence reveals that the Li_2_OHCl exhibit satisfying compatibility with liquid electrolyte; subsequently, lithium metal anode is protected with the *in situ* solidified buffer layer on anode interface by adding liquid electrolyte on the anode–SSE interface in the battery assembly; finally, the mechanism on how electrochemical performance improved by the buffer layer is discussed. The *in situ* welding strategy connects the anode and the SSE pellet, provides a uniform Li^+^ transport channel, and buffers the anode volume change. With the lower overpotential and improved cycle stability, symmetric lithium–metal batteries show novel cycling performance for 400 h and withstand the current density of 0.4 mAh cm^−2^; the battery with lithium–metal anode and LiFePO_4_ cathode illuminates significantly improved cycling endurance and capacity retention.

## Data Availability

The original contributions presented in the study are included in the article/Supplementary Material. Further inquiries can be directed to the corresponding authors.
