# Supplementary material for: Optimized Interfaces in Anti-Perovskite Electrolyte-Based Solid-State Lithium Metal Batteries for Enhanced Performance
Source: Front Chem. 2021 Dec 23;9:786956. doi: 10.3389/fchem.2021.786956 (PMC8733680; doi:10.3389/fchem.2021.786956)
Supplement: Supplementary file 1 [file DataSheet1.docx]

Supplementary Material

# Supplementary Tables

| **Solvent** | **Dipole moment (C·m)** | **B_p_ (℃)** | **Li_2_OHCl**  **compatibility*** |
| --- | --- | --- | --- |
| H_2_O | 1.85 | 100 | Incompatible |
| THF | 1.63 | 66 | Compatible |
| DMC | 0.76 | 90 | Compatible |
| DME | 1.15 | 85 | Compatible |
| DMF | 3.82 | 153 | Incompatible |
| DMSO | 3.96 | 189 | Incompatible |
| DOL | 1.50 | 75.6 | Compatible |
| ACN | 3.92 | 82.0 | Compatible |

**Supplementary Table 1.** Properties of common battery solvents and their compatibility with Li_2_OHCl.

*Compatibility Test: The solvent compatibility of Li_2_OHCl electrolyte has been evaluated by characterizing the sample through X-ray diffraction (XRD) and Fourier transform infrared (FT-IR) after solvent immersion and drying. The results are displayed in Supplementary Figure 2 and 3. It was revealed that the sample could keep the original Li_2_OHCl phase after exposure in THF, DMC, DME, DOL and ACN; however, it decomposed in H_2_O, DMF and DMSO.

#
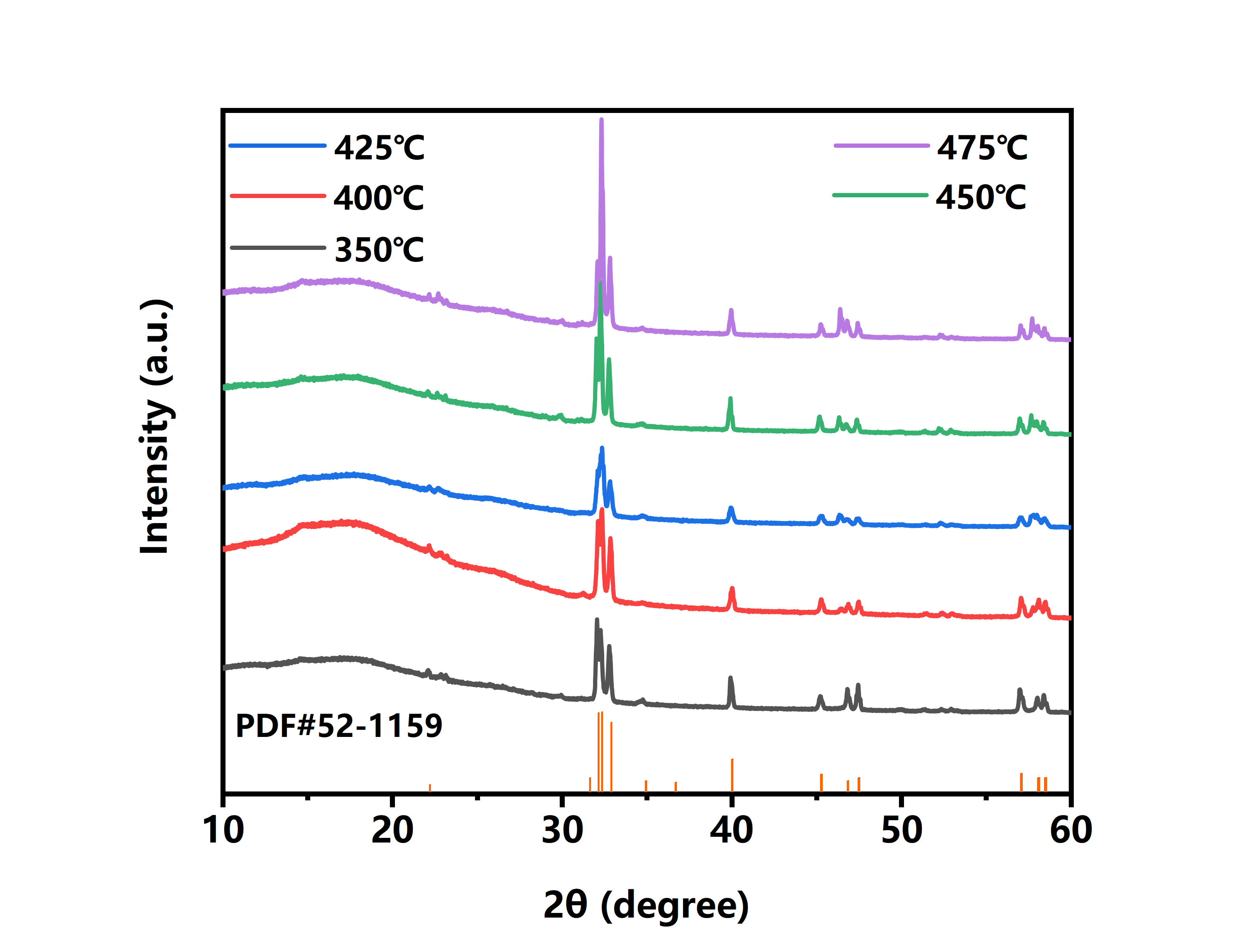
Supplementary Figures

**Supplementary Figure 1.** XRD patterns of the Li_2_OHCl powder in different calcining temperatures.

**Supplementary Figure 2.** XRD patterns of the Li_2_OHCl powder before and being soaked into various common battery solvents.


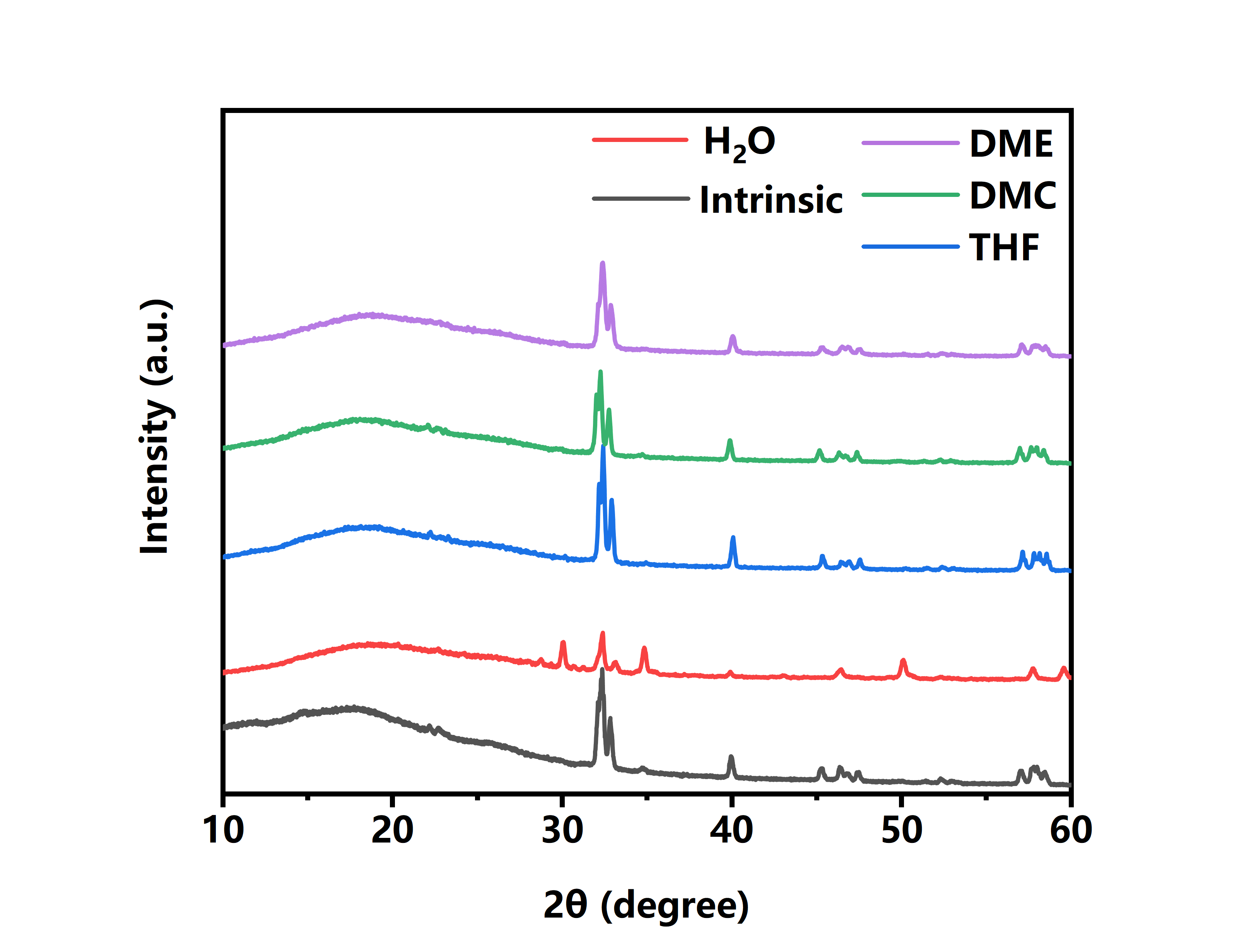

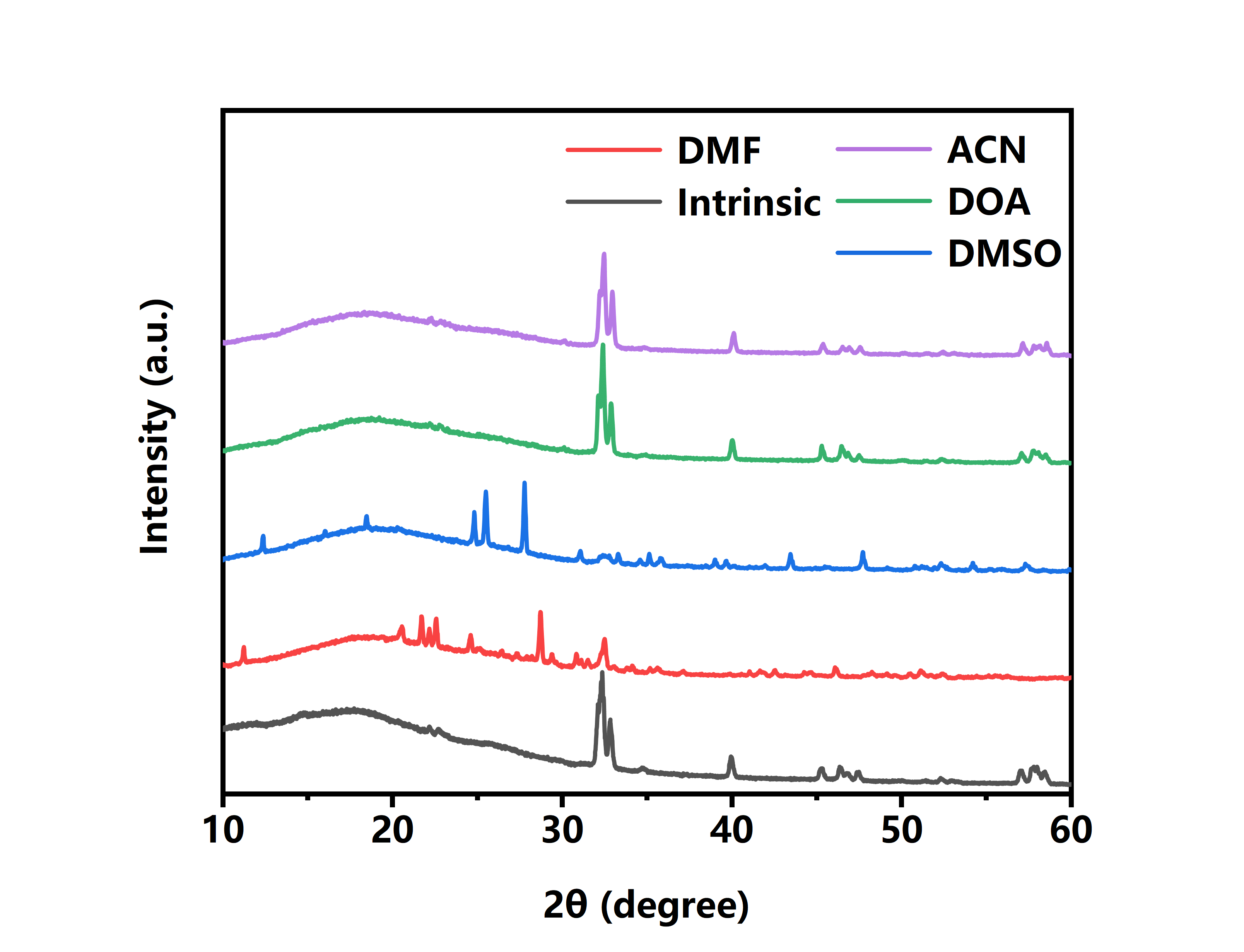


**Supplementary Figure 3.** IR patterns of the Li_2_OHCl powder before and after the soaking with various common battery solvents.
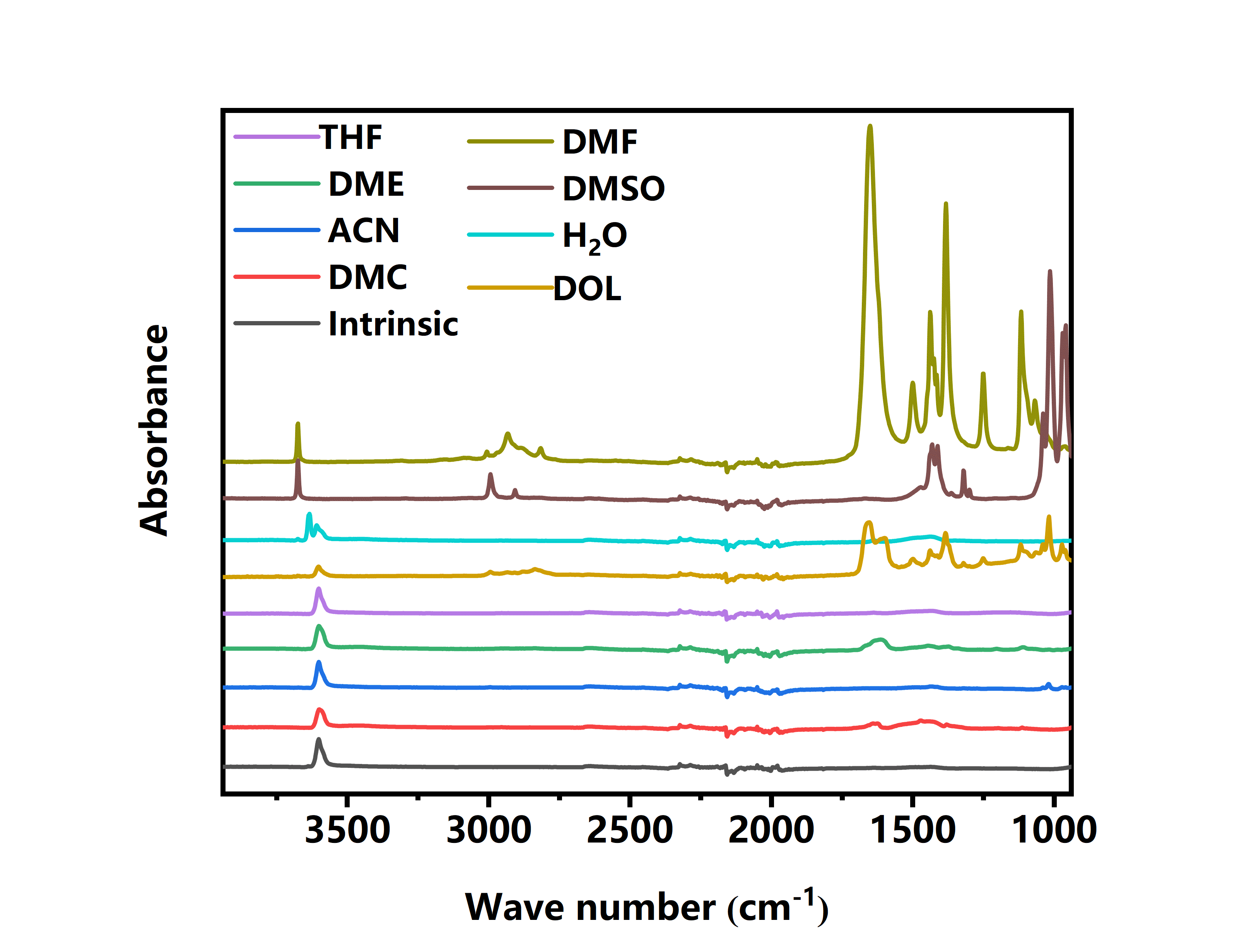


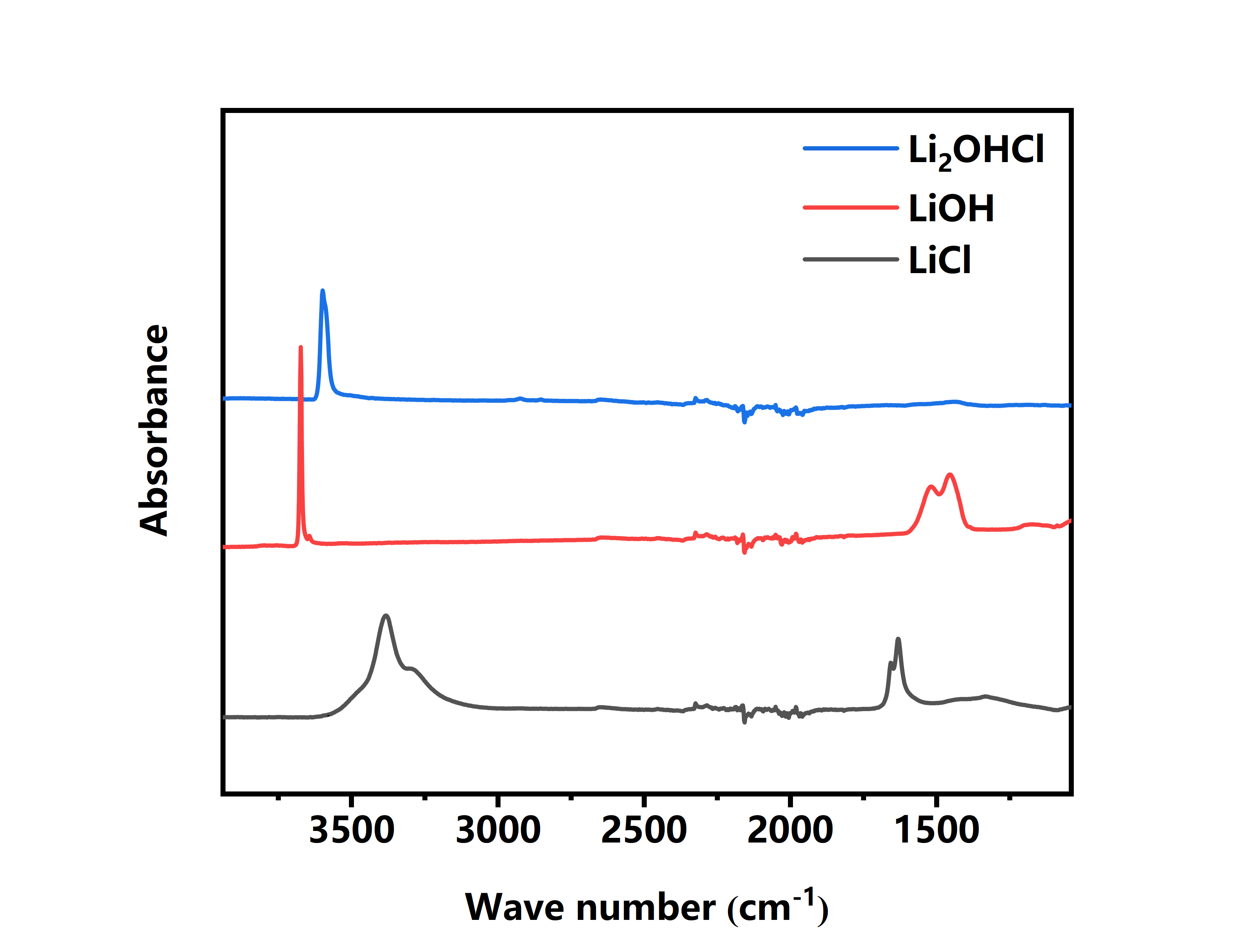
**Supplementary Figure 4.** IR patterns of the LiCl, LiOH, and Li_2_OHCl powder.

**
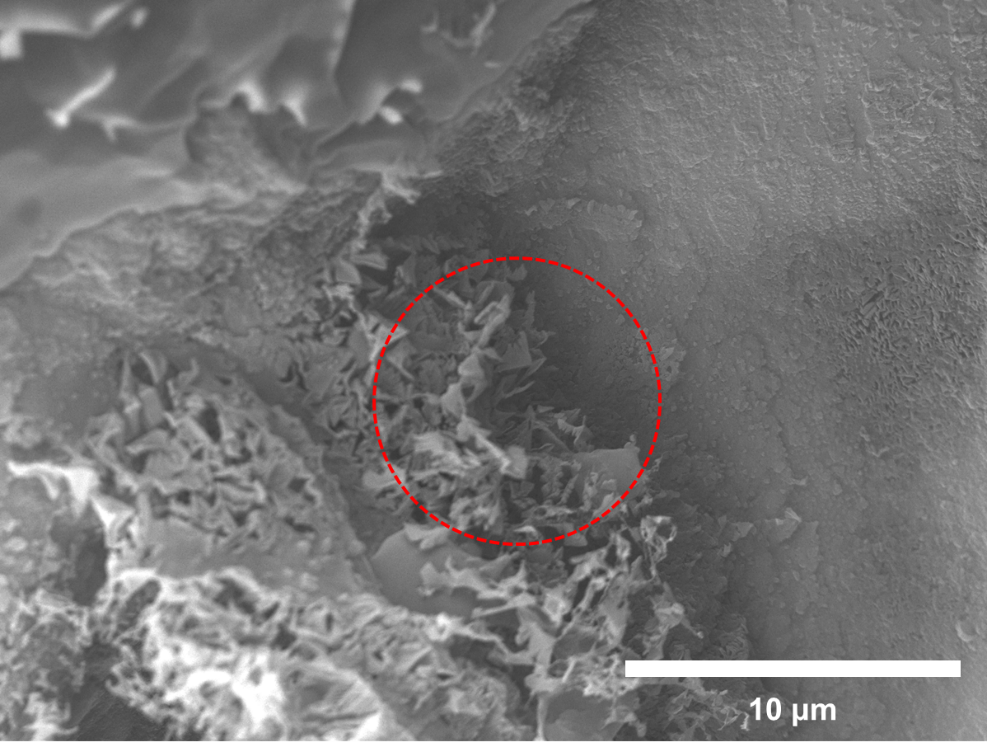
Supplementary Figure 5.** The morphology of the inner surface of a crack in a solid-state electrolyte pellet from a failed cell.

**
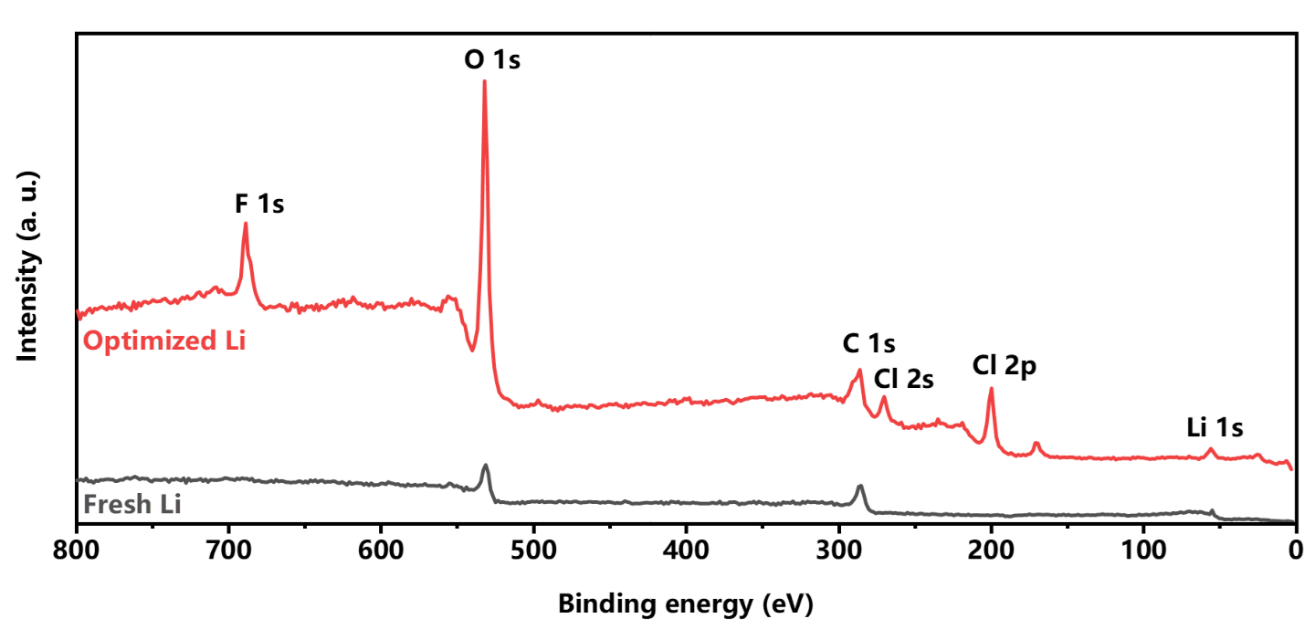
**
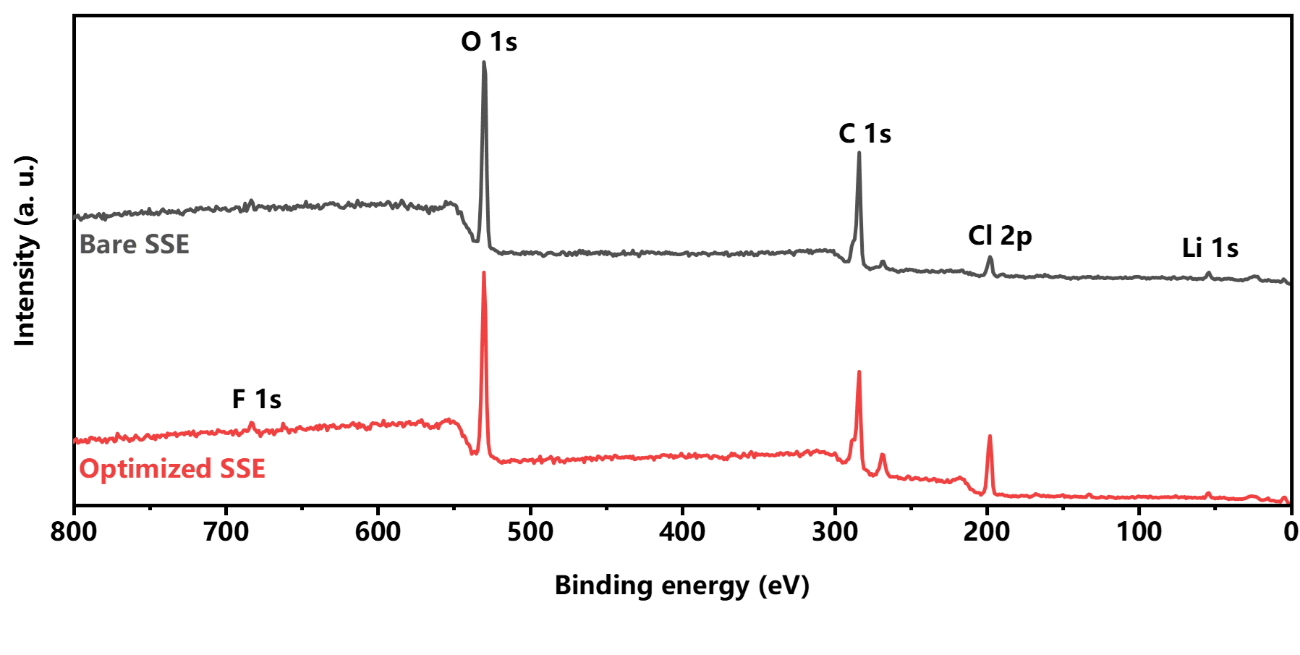
**Supplementary Figure 6.** XPS spectra of the SSE pellet and lithium metal anode.

**
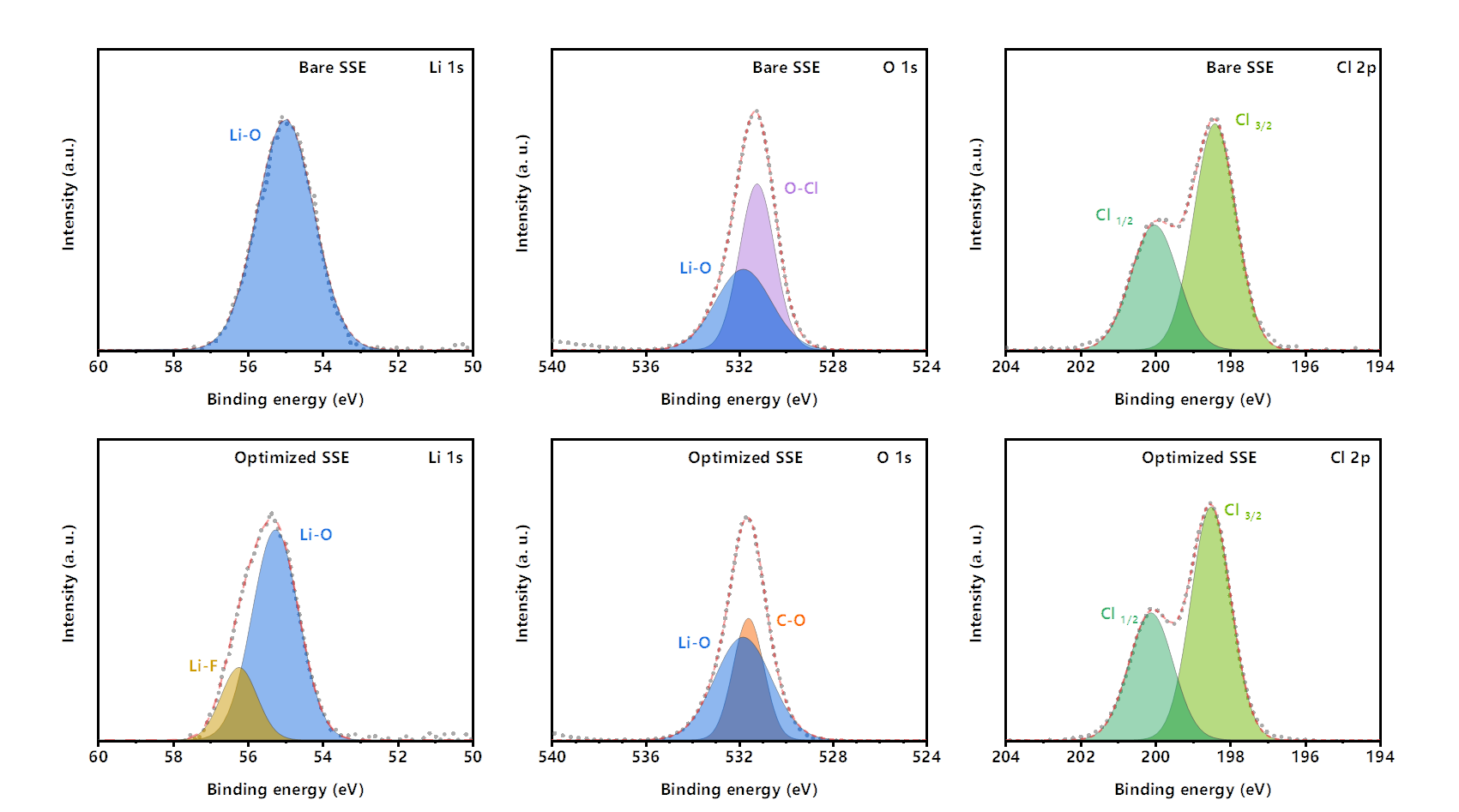
Supplementary Figure 7.** Li 2s, O 1s and Cl 2p XPS data and simulated peaks of the SSE pellet.
